# Temporal and Spatial Distribution Patterns of *Spodoptera frugiperda* in Mountain Maize Fields in China

**DOI:** 10.3390/insects13100938

**Published:** 2022-10-16

**Authors:** Yanyan He, Kun Wang, Guangzu Du, Qiong Zhang, Bin Li, Lin Zhao, Ping He, Bin Chen

**Affiliations:** 1 State Key Laboratory of Conservation and Utilization of Biological Resources of Yunnan, College of Plant Protection, Yunnan Agricultural University, Kunming 650201, China; 2 School of Agriculture, Yunnan University, Kunming 650500, China; 3 Plant Protection and Inspection Station of Qujing City, Qujing 655000, China; 4 Plant Protection and Inspection Station of Shizong County, Shizong 655700, China

**Keywords:** *Spodoptera frugiperda*, mountain maize, temporal distribution, spatial distribution

## Abstract

**Simple Summary:**

The global pest *Spodoptera frugiperda* (Lepidoptera: Noctuidae) colonized maize in Shizong, Qujing, Yunnan, China in 2019 and caused great economic loss. To monitor this pest and reduce the damage it causes, this study explored the temporal and spatial distribution of *S. frugiperda* in local fields from 2020 to 2021. The results showed that (1) there were two peaks in the density of eggs, larvae, and adults in both 2020 and 2021 throughout the maize growth period; (2) larvae of all instars were found in all growth stages and in different parts of maize, except 1st instar larva, which did not damage the male ears. However, the relative composition of different instars differed among growth stages and parts of maize; (3) larvae mainly damaged the heart leaf before the tasseling stage and the female ears after the tasseling stage; (4) early instar larvae were aggregated in mountain maize fields, while late instar larvae were distributed uniformly. This study provides monitoring data for *S. frugiperda* that can function as a guide for predictive models in integrated pest management programs.

**Abstract:**

*Spodoptera frugiperda* (Lepidoptera: Noctuidae) is a major pest of maize worldwide. This pest colonized maize in Shizong, Qujing, Yunnan, China in 2019. To explore the temporal and spatial distribution of *S. frugiperda* in local fields, “W” type 5-point sampling was performed from 2020 to 2021. The spatial distribution was analyzed using the aggregation index, Iwao’s regression, and Taylor’s power law. The temporal distribution showed two peaks for both 2020 and 2021 when the density of eggs, larvae, and adults was high throughout the maize growth period. Additionally, 1st and 3rd instar larvae were higher in number during the maize seedling, jointing, and spinning stages. Fourth to 6th instar larvae were higher in number after the tasseling stage. Additionally, the spatial distribution results showed that 1st to 3rd instar larvae were aggregated, while 4th to 6th instar larvae were uniformly distributed in mountain maize fields. This study provides monitoring data for *S. frugiperda* and clarifies the temporal and spatial distribution characteristics for larvae in mountain maize fields. Further, it also provides guidance for investigation into population dynamics and the development of predictive models for integrated *S. frugiperda* management.

## 1. Introduction

*Spodoptera frugiperda* J.E. Smith (Lepidoptera: Noctuidae), also known as the fall armyworm, is an important agricultural pest [1,2] that is native to tropical and subtropical areas of America [3,4]. The pest is affected by frequent international trade activities and the natural monsoon climate. *Spodoptera frugiperda* invaded Africa for the first time in 2016, and it has since spread to 44 African countries, where it poses a significant threat to food and nutrition security [2,5,6]. The pest invaded India in May 2018 [7], Myanmar in December 2018 [8], and Yunnan, China in January 2019 [9]. After the arrival in Yunnan, the pest quickly spread to most provinces in China within one year [10]. Recent studies have shown that the pest has spread to more than 100 countries throughout the Americas, Africa, Asia, and Oceania. *Spodoptera frugiperda* has become a major migratory pest, having a significant impact on crop production worldwide [10]. 

The significant economic impact caused by *S. frugiperda* is due to its wide host range, strong reproductive ability, and migratory ability [11,12]. There are more than 350 species of host plants for *S. frugiperda* [4,12,13,14]. The pest attacks maize, rice, sugarcane, wheat, soybean, and other crops [15,16,17]. However, *S. frugiperda* prefers maize, and it can cause a yield loss of 15–73% [2] and can complete one generation in a month without diapause and often causes remote outbreaks through long-distance migration [13,18]. Since *S. frugiperda* infestations started spreading in China, 98% of maize production areas in China have been severely damaged [10]. Yunnan is a place with a south tropical climate and is characterized by maize planting; it is the “first stop” for many major pests, such as *Nilaparvata lugens*, *Sogatella furcifera,* and *Cnaphalocrocis medinalis*, which migrate from Southeast Asia to China. Therefore, Yunnan is an important place for monitoring and predicting migratory pests [11,13]. 

Since the infestation of the *S. frugiperda* in China, Chinese plant protection scientists have systematically investigated and tracked *S. frugiperda* and developed a set of early warning and control strategies [19,20,21,22]. Global experts have also conducted significant research on the biological characteristics [23], damage characteristics [24], occurrence [25], spatial distribution pattern [13], sampling technology [13], flight ability [26,27], pesticide resistance [28], and other characteristics of this pest. Studies have shown that *S. frugiperda* larvae crawl into core leaves and produce silk in the wind [29]. Sometimes, the larvae can spread to other plants via adjacent leaves. Jiang et al. [30] found that Yunnan, Guangxi, Hainan, and Guangdong provinces were the most affected places, followed by Guizhou, Fujian, and Sichuan. The larvae of *S. frugiperda* were shown to be clustered in Brazilian maize fields [31] and randomly distributed in Colima, Mexico [32]. Sun et al. [13] conducted a study on maize fields in Dehong, Yunnan province, and found that the larvae of *S. frugiperda* were clustered. 

The study of the spatial and temporal occurrence of insects aids in the understanding of the interaction between insects and the environment and of insect population dynamics. Understanding these interactions can help to improve the accuracy of pest prediction, improving pest prevention and control. There have been some reports on the occurrence and damage [4,24] and spatial distribution patterns [13,32] of this species. Also, resistance of *S. frugiperda* populations changes with the use of insecticides [12]. Consequently, the distribution of and damage caused by *S. frugiperda* in the field might change over time. Therefore, many years of multi-point long-term monitoring are needed to provide timely and accurate guidance for the field management of this pest.

In this study, we monitored and investigated the population dynamics of adults, eggs, larvae, and pupae of *S. frugiperda* throughout the growth period of mountain maize in Shizong, Qujing, and Yunnan provinces (China) for two consecutive years from 2020 to 2021. This study identified the spatial distribution patterns and sampling techniques associated with the larvae. Lastly, this study provides timely data on the occurrence of *S. frugiperda* in maize fields and guidance for controlling this pest in these fields.

## 2. Materials and Methods

### 2.1. Age-Stage Temporal Distribution and Occurrence Regularity of S. frugiperda in 2020–2021

#### 2.1.1. Test Sites

The test site was in Dashanjiao Village, Dazhai Village Committee, Longqing Township, Shizong County, Qujing City, southeast Yunnan province, China (altitude: 1340.25 m, 104°4′54″ E, 24°29′28″ N). The maize was planted in a mountain field, and the soil was clay with medium fertility. Three maize fields with consistent field management conditions were selected. The selected fields were 400 m^2^ each, and all maize varieties were Zhidan No. 8. Before the planting of maize in these fields, wheat had been grown. The maize was sown on April 15 and harvested on 22 September 2020. In 2021, the maize was sown on 28 May and harvested on 28 September with no pesticide application throughout the two-year growth period. 

#### 2.1.2. Occurrence of *S. frugiperda* Larvae

From May to October 2020 and from June to October 2021, the “W” five-point sampling method was adopted at the maize seedling stage, and “ladder” type five-point sampling was adopted after the tasseling stage [33]. In this study, five samples with 10 maize plants per sample were selected and surveyed once per day for seven days. Different parts of the plant were checked, including the front, back, and base of the leaves, heart leaves, stems, tassel, filaments, female ears, and other parts. The number of *S. frugiperda* larvae in different parts of the maize plant was recorded. Additionally, the rate of plant damage (%) and the number of pests per 100 maize plants were determined and recorded.

#### 2.1.3. Population Dynamics of Adult *S. frugiperda*

The population dynamics of adult *S. frugiperda* were determined using captured male moths from sex pheromone traps. The sex pheromone traps were of the bucket type and were produced by Beijing Zhongjie Sifang Biological Technology Co., Ltd. (Beijing, China). The lure core was specific for *S. frugiperda* using sex pheromones. The lures were kept in a refrigerator at 4 °C in accordance with the manufacturer’s instructions, and the sex pheromone was replaced every 40 days. Three traps were placed in each field. The traps were placed one meter from the ground in the seedling stage and 20 cm higher than the plant canopy in the other stages. The traps were placed vertically, and the distance between the traps was at least 50 m. From June to October 2020, and from June 2021 to January 2022, the captured adults were collected and recorded every seven days [34].

#### 2.1.4. Number and Location of Egg Masses of *S. frugiperda*

The number and location of *S. frugiperda* eggs were determined using “W” type sampling at five points in each field [33]. Ten maize plants were randomly sampled at each point, and their stalks and the front, back, and base of the leaves were checked every six days. The number of eggs on the sampled maize plants and the spawning location were recorded.

#### 2.1.5. Number and Location of *S. frugiperda* Pupae

According to Liu et al. [35], to determine the number and location of *S. frugiperda* pupae, 5 points (each 1 m^2^) were randomly selected in the field where the larval density was high. The numbers and location of pupae in the soil at a depth of 10 cm were recorded at each point.

### 2.2. Spatial Distribution of S. frugiperda Larvae in 2020–2021

#### 2.2.1. Spatial Distribution Type

The spatial distribution of *S. frugiperda* larvae was analyzed using the aggregation index method Iwao’s regression analysis and Taylor’s power method [13,36,37]. The variance (*S*^2^) and mean larval density (*m*) were calculated using the number of larvae in each sample. The spatial distribution parameters were calculated using the formula shown in Table 1.

#### 2.2.2. Sampling Techniques

In accordance with Iwao [13] and Sun et al. [42], sampling was performed using the theoretical and sequential sampling technique models. For the theoretical sampling model, we used the equation:

(1) $$N=(\frac{t^{2}}{D^{2}})(\frac{\alpha +1}{m}+\beta -1)$$

where *N* is the optimal theoretical sampling number; *t* is the distribution value at a certain confidence level; *D* is the allowable error; *α* is the intercept; and *β* is the slope in Iwao’s regression equation. For the sequential sampling technique model, we used the equation:

(2) $$T_{I\mathrm{wao}(n)}=nm_{0}\pm t\sqrt{n[(\alpha +1)m_{0}+(\beta -1){m_{0}}^{2}]}$$

where the lower limit *T*_2 *(n)*_ is the value calculated by the minus sign, the upper limit *T*_1 *(n)*_ is the value calculated by the plus sign, *m_0_* is the control index, *n* is the sampling number in the field, *t* is the distribution value under the corresponding confidence degree, and *α* and *β* are the same as in the theoretical sampling calculation model. If the insect quantity is greater than the upper limit when sampling, the population density is higher than the control index. On the other hand, if the insect quantity is less than the lower limit, the population density is lower than the control index, and sampling needs to be continued.

## 3. Results

### 3.1. Developmental Stage Temporal Distribution of S. frugiperda in 2020–2021 in the Mountain Maize Field

**Figure 1:**
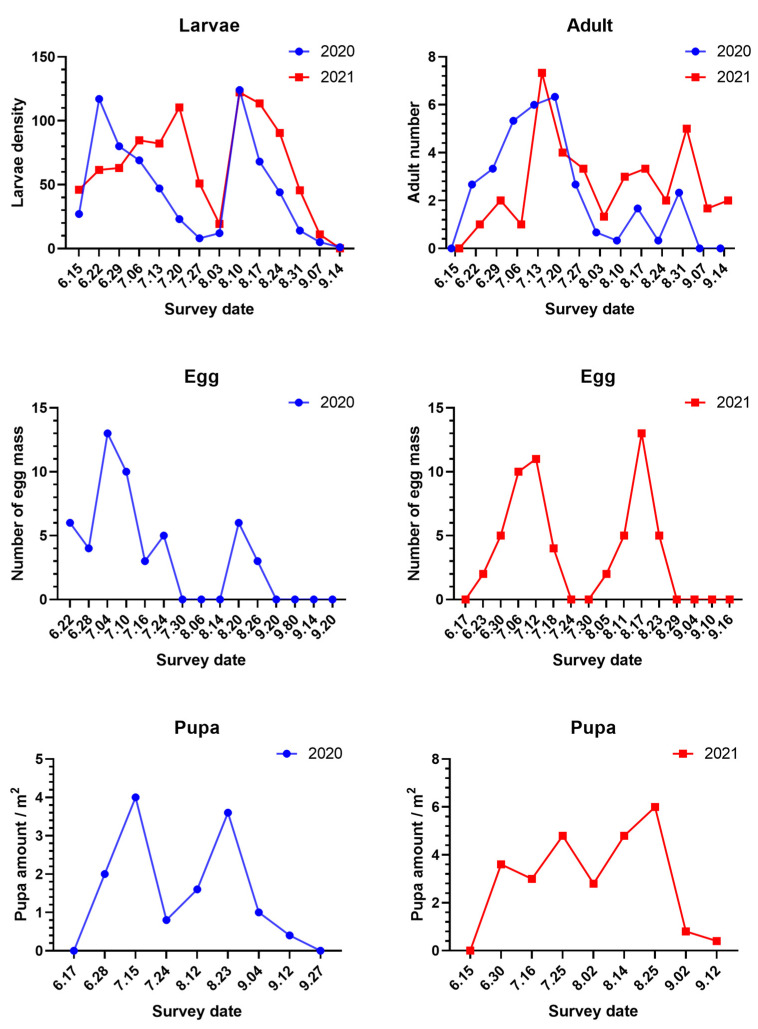
The population dynamics and occurrence of *Spodoptera frugiperda* at different stages in mountain maize fields. The larval density expresses the number of larvae per one hundred maize plants, the adult number indicates the number of adults per trap, and the number of egg masses is the number of egg masses per hundred maize plants.

The dynamics of *S. frugiperda* larvae in the mountain maize field in 2020 and 2021 showed that the larvae could damage maize throughout all planting stages, as shown in Figure 1. The results showed that there were two peaks in the number of *S. frugiperda* larvae during the growth period of maize. In 2020, larval damage showed an increasing trend from 15 June to 22 June, and the first peak was reached on 22 June (density was 117.00 larvae/100 plants; jointing stage). From 22 June to 3 August (powdering stage), the damage caused by larvae began to decline, and it rose again from 3 August to 10 August (spinning stage) and reached the second peak value on 10 August. The peak in August was the highest value for the whole year. The results showed that the larval population kept decreasing after the spinning stage. In 2021, the number of larvae started to increase from 15 June to 20 July and reached the first peak on 20 July, when the maize was entering the flare opening stage. Afterwards, the number decreased sharply and reached the lowest point on 3 August. The number of larvae began to rise again after 3 August and reached the second peak on 10 August (122.20 larvae/100 plants), when the maize entered the spinning stage. The second peak in 2021 was the highest number for the entire year. Since then, the larval population has been on a downward trend. There was no difference between the number of larvae in the two consecutive years except for in the first peak of each year.

The trend in the number of adults of *S. frugiperda* collected in two successive years was basically the same, as shown in Figure 1. The number of adults trapped began to rise starting in 22 June. The number continued to rise through mid to late July. The numbers recorded in July were the highest for the whole year (with a peak of 20 adults/trap on 20 July 2020, and a peak of 22 adults/trap on 13 July 2021). After July in each year, the number of adults started to fall, decreasing to one adult/trap and four adults/trap on 10 August 2020, and 3 August 2021, respectively. After that, the number of adults in the traps increased and reached the second peak on 31 August. However, the number further fell to zero adults/trap on 7 September 2020, and six adults/trap on 14 September 2021.

The number of *S. frugiperda* egg masses had two peaks between 22 June and 20 September 2020 (Figure 1). The highest number of *S. frugiperda* egg masses was recorded on 4 July (trumpet period) with a density of 13 masses/100 plants, and the second peak was on 20 August (pustulation stage) with a density of six masses/100 plants, as shown in Figure 1. In 2020, eggs were found on plants from 22 June to 24 July and from 20 August to 26 August, but no eggs were found on maize between 30 July and 14 August or between 2 September and 20 September. From 17 June to 16 September 2021, there were two peaks in the number of egg masses in the field. The first peak was on 12 July (trumpet stage). The second peak was on August 17 (pustulation stage). However, no eggs were recorded from 24 July to 30 July or from 29 August to 16 September. Thus, these 2-year studies on *S. frugiperda* oviposition found two peak periods. The first peak was in early July, when maize was in the trumpet stage, and the second peak was in late August, when the maize was in the pustulation stage. No eggs were found on maize plants from the end of July to the beginning of August (tasseling, powdering stage) and after the end of August (milk ripening, waxy ripening stage). 

Additionally, females preferred to lay eggs on the backs of leaves during the jointing stage with a distance of 0–0.5 cm between egg masses and the midvein (Table S1). Egg masses were most frequently located at a distance of 0–10 cm from the main stem, and 42.68% of the egg masses had between 50 and 100 eggs. The incubation time was mostly two days. There was no significant difference in the number of egg masses with and without villi. In the field, some eggs were parasitized by egg parasitoids, as shown in Table S1.

The pupal density in the field in 2020 showed two peaks on 15 July (flare opening stage) and 23 August (pustulation stage) with pupa quantities of 4.0 and 3.6 pupae/m^2^, respectively (Figure 1). In 2021, there were three peaks on 30 June (trumpet stage), 25 July (tasseling stage), and 25 August (pustulation stage), with populations of 3.6, 4.8, and 6 pupae/m^2^, respectively, as shown in Figure 1. In 2021, the number of pupae in fields was higher than in 2020, and pupation depth of 2–4 cm was the most common, followed by those with a depth of 4–6 cm. In 2020 and 2021, the number of pupae at a depth of 2–4 cm was 36 and 59, respectively, as shown in Figure S1.

### 3.2. Spatial Distribution Pattern of S. frugiperda in 2020–2021 in Mountain Maize Fields

#### 3.2.1. Occurrence and Damage of *S. frugiperda* in Mountain Maize Fields in 2020–2021 

**Figure 2:**
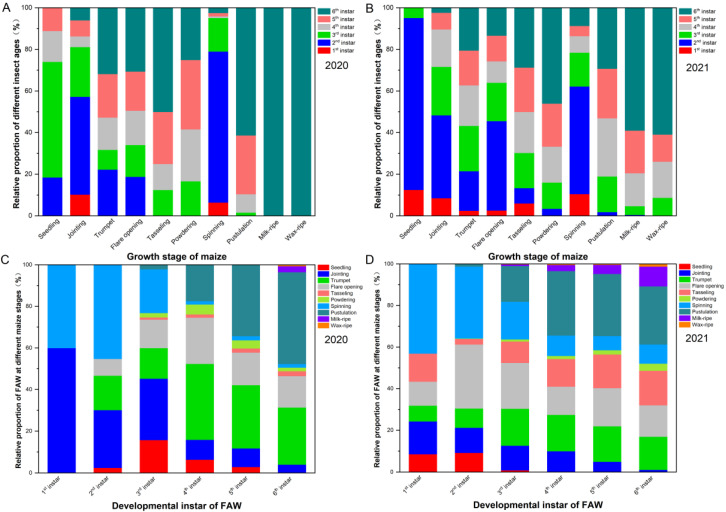
The relative damage proportions of *S. frugiperda* larvae (1st instar to 6th instar) in mountain maize fields across growth stages in (**A**) 2020 and (**B**) 2021, and the relative damage proportion of *S. frugiperda* larvae at different maize stages (from seedling stage to waxy stage) in mountain maize fields across the development instar of *S. frugiperda* larvae in (**C**) 2020 and (**D**) 2021.

We found that the 2nd and 3rd instars were the most common larval stages in the maize seedling stage (Figure 2A,B). At this stage of crop growth, it was found that the pest damaged both the heart and young leaves. At the jointing stage, the larvae were mainly in the 2nd and 3rd instars, accounting for 37.47% and 27.36% of the total pest population, respectively. Further, it was found that the feeding position of the pest at the jointing stage of maize was similar to that in the seedling stage. The proportions of 2nd, 5th, and 6th instar larvae in the trumpet stage were 20.46%, 23.35%, and 31.88 % of the total pest population, respectively, and all instars of the pest appeared at this stage with the exception of 1st instar larvae, which mainly fed on the heart leaves of the crop. 

For the flare opening stage, the heart leaf was the most commonly damaged part of the crop; 6th instar larvae had the largest population, and 1st instar larvae were not found (Figure 2A). In the tasseling stage, 6th instar larvae accounted for the largest population, and 1st and 2nd instar larvae were not found. The main feeding positions of the larvae at the tasseling stage were the male ears and young leaves. In the powdering stage, 4th and 5th instar larvae accounted for the largest population and damaged the young leaves and the male and female ears of the crop. 

In the spinning stage, 2nd instar larvae accounted for the largest population (Figure 2A). In addition, larvae of all instars appeared at this stage of maize growth and mainly fed on the female ears. In the pustulation stage, it was found that 5th and 6th instar larvae fed on female ears and damaged the maize grains. At the milk and waxy ripening stages, the young parts of maize decreased, and the pest population density was low. However, it was evident that 6th instar larvae were most abundant.

In 2021, *S. frugiperda* larvae on maize at the seedling stage were all young, and larvae of the 2nd instar made up the highest proportion, accounting for 82.02% (Figure 2B). Further, it was evident that the heart and young leaves were harmed at this stage of growth. At the jointing stage, larvae of all instars appeared, and 2nd instar larvae accounted for 38.20% of the total population. In addition, the feeding site of the pest at the jointing stage was the same as in the seedling stage. In the trumpet stage, except for 1st instar larvae, there were insignificant differences among the larval instars, which mainly fed on the heart leaves of the crop. 

The population of 2nd instar larvae in the flare opening stage was the largest, and there were no significant differences in the population densities of larvae of other instars, except for 1st instar larvae, which mainly fed on the heart leaves of the crop (Figure 2B). At the tasseling stage, the larvae of the 4th, 5th, and 6th instars represented 19.77%, 21.37%, and 28.87% of the total population, respectively. At this stage, the larvae fed on the tassels and young leaves of the crop. The population of 6th instar larvae at the powdering stage was large in proportion and damaged the young leaves and male and female ears. In the spinning stage, 2nd instar larvae accounted for 51.56% of the total population, and larvae mainly fed on the female ear filaments. In the pustulation stage, the aged larvae fed on the female ears and damaged the maize grains (Table S2). At the milk ripening stage, 6th instar larvae were mainly found in the field, accounting for 57.48% of the total population. At the waxy stage, the young parts of maize were reduced, and 6th instar larvae were most abundant, accounting for 62.22% of the total population.

First instar larvae caused damage in the jointing and spinning stages in 2020, while they caused damage from seedling to tasseling stages and in the spinning stage in 2021 (Figure 2C,D). There were fewer damaged growth stages by 2nd and 3rd instar larvae in 2020 than in 2021. Fourth to 6th instar larvae caused damage in all growth stages, except for in the seedling stage, in 2021. However, 4th to 5th instar larvae damaged maize from the seedling stage to the pustulation stage in 2020. For larvae of all instars, the relative proportion in the tasseling stage in 2020 was less than in 2021.

In 2021, 1st instar larvae were not distributed on the male ears, but all instar larvae were distributed among different parts of maize crops (Table S3). Further, there were significant differences in the proportions of larvae on different parts of the maize, such as the heart leaves, stems (leaves), male ears, and female ears, but 1st instar larvae were an exception to this. From the 1st to 6th instars, larvae were mainly distributed in the heart leaves and female ears. The proportion of early instar larva (between the 1st and 3rd instars) on the stems (leaves) was over 20%, while the proportion of late instar larvae (between the 4th to 6th instars) on the stems (leaves) was less than 20% of the total population. The proportion of larvae between the 1st and 6th instars on the spike was less than 20% of the total population. The distributions of the number of larvae in the different parts of maize from high to low were heart leaves > female ears > stem leaves > male ears (Table S3). 

#### 3.2.2. The Density and Spatial Distribution Patterns of *S. frugiperda* Larvae in Mountain Maize Fields 

In 2020, the results of all aggregation index methods showed that the larval distribution was uniform among the trumpet, tasseling, and milk ripening stages of maize. However, it was evident that the larvae were aggregated at the other growth stages of maize (Table 2). 

According to the Iwao formula, the linear regression equation is

(3) m*=0.36+1.31m,

*R* = 0.52, *α* = 0.36, *β* = 1.31 > 1.

In addition, the relative Taylor’s formula is

(4) $$\mathrm{lg}s^{2}=1.11lgm+0.19,$$

*R* = 0.96, *lga* = 0.19 > 0, *b* = 1.11 > 1.

Therefore, the results of the aggregation index tests were consistent with those of the Iwao and Taylor’s regression analyses, indicating that the spatial distribution of *S. frugiperda* larvae in the mountain maize fields was aggregated in 2020, and the aggregation degree was density-dependent.

In 2021, all aggregation indices of larvae in the powdering stage had values of less than 1, and the average crowding degree (M*), agglomeration index (M*/m), and diffusion coefficient (*C*) of the other growth stages had values of greater than 1 (Table 3). The diffusion indices (*I*) of the trumpet and pustulation stages, as well as the negative binomial distribution (*K*) of both the seedling and jointing stages, had a value of less than 1. On the other hand, the diffusion index (*I*) and negative binomial distribution (*K*) for the other stages had values higher than 1. Further, the Kuano index (*C_a_*) had a value greater than 1, although only in the seedling and jointing stages. Therefore, the spatial distribution of larvae in mountain maize fields showed an aggregated distribution in 2021. According to the Iwao formula, the linear regression equation is m* = 0.47 + 1.34 *m* (*R* = 0.82), *α* = 0.47, *β* = 1.34 > 1. Furthermore, the relative Taylor’s formula is *lgS*^2^ = 1.39 *lgm* + 0.21 (*R* = 0.93), *a* = 0.21 > 0, *b* = 1.39 > 1. Therefore, the results of the Iwao and Taylor’s linear regression models are consistent with the aggregation index test, which also showed that the spatial distribution of larvae in the mountain maize field was aggregated in 2021. The results of the investigation conducted over two years showed that the larval distribution pattern of *S. frugiperda* in maize fields in southeastern Yunnan was aggregated.

In addition, the distribution pattern of early instar (1st–3rd) larvae of *S. frugiperda* was also aggregated in 2020 and 2021 (Tables S4 and S5). On the other hand, the late instar (4th–6th) larvae were evenly distributed in 2020 and 2021 (Tables S6 and S7). In 2020, the average density of early-instar larvae at the spinning, jointing, and other growth stages of maize were higher than 1, close to 1, and less than 1 larva/plant, respectively. In 2021, the average density of early-instar larva at the seedling, jointing, trumpet, and spinning stages was greater than 1 larva/plant, whereas the average density of late-instar larva at other growth stages was less than 1 larva/plant. 

In 2020, the population density of larva in the early instar stages was not high in all growth stages of maize, and the average density was less than 1 larva/plant (Table S5). In 2021, the population density of the late-instar larvae was higher in the trumpet, flare opening, tasseling, and pustulation stages of maize growth, and the average density was greater than 1 larva/plant (Table S7). The average population density of late-instar larvae was less than 1 larva/plant at the jointing, powdering, and spinning stages. It was also noted that there were no late-instar larvae at the seedling stage.

#### 3.2.3. Theoretical and Sequential Sampling Numbers of *S. frugiperda* Larvae at Different Densities 

The coefficients from the Iwao m*-M regression model (*α* = 0.88, *β* = 1.49; *α* = −0.02, *β* = 0.82; *α* = 0.36, *β* = 1.31) for the early-/late-/total instar larvae of *S. frugiperda* in 2020 were substituted into the formula. The theoretical sampling number models for the early-instar, late-instar, and total instar larvae (respectively) were as follows (Table 4):

(5) $$N=(\frac{3.84}{D^{2}})(\frac{1.88}{m}+0.49)\;,$$

(6) $$N=(\frac{3.84}{D^{2}})(\frac{0.98}{m}-0.18),$$

(7) $$N=(\frac{3.84}{D^{2}})(\frac{1.36}{m}+0.31)$$

According to the results of previous studies [13], prevention and control measures should be taken against pests when the larval population density reaches 0.33 larvae per plant in maize fields. The coefficients from the Iwao m*-M regression model (*α* = 0.88, *β* = 1.49; *α* = −0.02, *β* = 0.82; and *α* = 0.36, *β* = 1.31) for the early-/late-/total instar larvae of *S. frugiperda* in 2020 were substituted into the sequential sampling model

(8) $$T_{\mathrm{Iwao}(n)}=nm_{0}\pm t\sqrt{n[(\alpha +1)m_{0}+(\beta -1){m_{0}}^{2}]}$$

The sequential sampling models for the early-instar, late-instar, and total instar larvae (respectively) were as follows:

(9) $$T_{\mathrm{Iwao}(n)}=0.33n\pm 1.96\sqrt{0.6738n},$$

(10) $$T_{\mathrm{Iwao}(n)}=0.33n\pm 1.96\sqrt{0.3038n},$$

(11) $$T_{\mathrm{Iwao}(n)}=0.33n\pm 1.96\sqrt{0.4826n}$$

Sequential sampling intervals of the larvae under different sample numbers (*n* = 60, 70, 80, …, 200) are listed in Table 5. 

Similarly, by substituting the responding coefficient of regression models of the early-/late-/total instar larvae in 2021 into the theoretical and sequential sampling models, the best theoretical and sequential sampling models for early-, late-, and total instar larvae were respectively obtained, as follows:

(12) $$N=(\frac{3.84}{D^{2}})(\frac{3.2575}{m}+0.2874),$$

(13) $$N=(\frac{3.84}{D^{2}})(\frac{1.008}{m}-0.0644),$$

(14) $$N=(\frac{3.84}{D^{2}})(\frac{1.4718}{m}+0.3370);$$

(15) $$T_{\mathrm{Iwao}(n)}=0.33n\pm 1.96\sqrt{1.1063n},$$

(16) $$T_{\mathrm{Iwao}(n)}=0.33n\pm 1.96\sqrt{0.3257n},$$

(17) $$T_{\mathrm{Iwao}(n)}=0.33n\pm 1.96\sqrt{0.5224n}$$

The theoretical optimal sampling number *N* and sequential sampling intervals are listed in Table 6 and Table 7.

## 4. Discussion

Globally, *S. frugiperda* is listed as one of the top 10 plant pests by the Centre for Agriculture and Bioscience International. The rapid spread of *S. frugiperda* has seriously threatened maize production and caused major economic losses worldwide [43]. The first location of *S. frugiperda* infestation in China was in Yunnan province. Yunnan is adjacent to multiple Southeast Asian countries, and its unique climate and geographical location provide favorable conditions for the invasion, annual reproduction, and northward spread of the pest. Therefore, there is an urgent need to strengthen the monitoring, early warning, prevention, and control of *S. frugiperda* in Yunnan. This may reduce local economic losses and the extent of northward migration of *S. frugiperda*. The temporal occurrence and spatial distribution of these insects reflects the interaction between individual insects and the environment. Understanding the regularity of temporal and spatial distributions of pests is crucial for planning control strategies, developing effective sampling plans, and predicting pest damage [44]. Since April 2019, *S. frugiperda* has successfully colonized Shizong, Qujing, and Yunnan provinces [45]. To understand the occurrence dynamics of *S. frugiperda* in the area and provide guidance for its control, the present study investigated the occurrence and spatial distribution pattern of *S. frugiperda* in mountain maize fields in Shizong, Qujing, and southeast Yunnan provinces from 2020 to 2021. 

The results of the current study showed that the larvae of *S. frugiperda* are harmful to maize immediately after the plant is fully grown, and the larvae prefer maize in the planting area over other plants. It is evident that *S. frugiperda* populations increased with an increase in temperature and with the growth of maize, and there were two annual peaks for *S. frugiperda* larvae in the trumpet stage and the spinning stage. Before the tasseling stage, the larvae mainly affected the heart leaves, but after the tasseling stage, they mainly affected the female ears. The relative proportion of early-instar larvae was higher before the tasseling stage than after the tasseling stage (except in the spinning stage). Therefore, it is evident that spraying the heart leaves of maize before the tasseling stage and spraying the ears after the tasseling stage could effectively control the pest. This is consistent with the findings of a study conducted by Han et al. [46], which found that *S. frugiperda* larvae mainly harmed the heart leaves before the jointing stage and the female ears after the spinning stage. In the different growth periods of maize, it was noted that larvae preferred feeding at the jointing, flare opening, and spinning stages, and the distributions of larvae in the maize plants were as follows (from high to low quantity): heart leaf > female ear > stem (leaf) > tassel. This showed that the pest favors the heart leaves and female ears, which are young parts of maize plants that are critical for the growth as well as the development of maize; this may be one of the reasons why the pest is significantly harmful. Previous studies conducted by Tang et al. [47] and Mutyambai et al. [48] found that *S. frugiperda* larvae had a significant preference for the tender parts of plants. In addition, the composition and structure of larvae were different at different growth stages of maize. It was evident that the number of early-instar larvae was higher at the seedling, jointing, trumpet, and spinning stages. However, the proportions of late-instar larvae in the tasseling, powdering, pustulation, milk ripening, and waxy ripening stages were significantly higher, which is consistent with the results of studies conducted by Qin et al. [49] in Guangxi province and Lu et al. [50] in Hainan province.

It is noted that the timely prevention and control of early-instar larvae should be strengthened in the seedling, jointing, flare opening, and spinning stages of maize to reduce the population size. In addition, pesticides with greater toxicity against early-instar larvae or with strong ovicidal activity should be used at the seedling/jointing/flare opening/spinning stages. Furthermore, together with the previous findings, the degree of damage to maize and the population density of *S. frugiperda* were found to relate to the growth period, variety, and location of maize, the age of the pest, the season, field temperature, light, region, plant species in the nearby habitat, as well as to local pest management measures [13,46,48]. 

The spatial distribution of pests provides a basis for planning control strategies, developing effective sampling plans, and predicting damage. The present study found that, in 2020 and 2021, *S. frugiperda* larvae were aggregated in mountain maize fields in Shizong, Yunnan, China. The results of previous studies on spatial distribution patterns conducted by Farias et al. [31], Serra et al. [51], and Sun et al. [13] also showed that the larvae of *S. frugiperda* were aggregated in Brazil, Argentina, and Dehong (Yunnan, China), but they were randomly distributed in Mexico [32]. However, it was found that the spatial distribution patterns of early- and late-instar larvae differed. The late-instar larvae were mainly uniformly distributed, whereas the younger larvae were clustered. A similar finding was found for the distribution of *S. frugiperda* in wheat in a study performed by Yang et al. [15]. This could be because the late-instar larvae have a habit of killing each other [52]. Moreover, in this study, most of the larvae were early-instar larvae from the seedling stage to the flare opening stage, and the population was clustered, whereas from the tasseling stage to the waxy ripening stage, there were mostly late-instar larvae (except in the spinning stage), and the pest population was evenly distributed, showing that the growth stages of the plant influenced the spatial distribution. Based on the field distribution of *S. frugiperda* over three consecutive years, Mitchell & Fuxa [53] found that different years and different population densities cause different types of distributions of *S. frugiperda*. 

It is evident that the spatial distribution of *S. frugiperda* is correlated with its spawning habits, spreading ability, host phenology period, and population density. It has been found that the source base of local pests and the number of immigratory pest sources can increase the population density, thus affecting the spatial distribution [15]. The current study only analyzed the spatial distribution of *S. frugiperda* larvae from a single variety of maize, and the spatial distribution patterns of larvae under different climatic conditions, soil, planting density, maize varieties, and other factors were not studied. Therefore, there is a need to strengthen the analysis of the spatial distribution and sampling technology of the larvae in different types of maize fields to validate and optimize the model and better guide the prevention and control of *S. frugiperda*.

## 5. Conclusions

In conclusion, this study identified two peaks of larval and adult occurrence of the *S. frugiperda* in mountain maize fields in 2020 and 2021. Before the tasseling stage, the pest mainly damaged the heart leaves, while after the tasseling stage, it mainly damaged the female ears. In terms of distribution, *S. frugiperda* larvae were mostly found to be aggregated in the maize field, and the distribution varied with the age of the larvae and the growth period of maize.

## Figures and Tables

**Table 1:** Formulas used to show the spatial distribution type.

Method	Formula and Reference	Distribution Type
Aggregation index	Mean crowding intensity (M*) $M^{*}=\bar{x}+S^{2}/\bar{x}-1$ (Lloyd, 1967) [38]	M* < 1, uniform distribution M* = 1, random distribution M* > 1, aggregated distribution
	Index of clumping (*I*) $I=S^{2}/\bar{x}-1$ (David and Moore, 1954) [39]	I < 0, uniform distribution I = 0, random distribution I > 0, aggregated distribution
	Clustering index (M*/M) $M^{*}/\bar{x}$ (Lloyd, 1967) [38]	M*/M < 1, uniform distribution M*/M = 1, random distribution M*/M > 1, aggregated distribution
	Kuno Index (*C_A_*) $C_{A}=\left(S^{2}/\bar{x}-1\right)/\bar{x}$ (Cassie, 1967) [40]	C_A_ < 0, uniform distribution C_A_ = 0, random distribution C_A_ > 0, aggregated distribution
	Morisita diffusion index (*C*) (Poisson distribution coefficient) $C=S^{2}/\bar{x}$	C < 1, uniform distribution C = 1, random distribution C > 1, aggregated distribution
	Water’s negative binomial distribution K value (*K*) $K=\bar{x}/(S^{2}/\bar{x}-1)$ (Waters, 1959) [41]	K < 0, uniform distribution K = 0, random distribution K > 0, aggregated distribution
Iwao regression analysis $m^{*}-m$	$M^{*}=\alpha +\beta \bar{x}$ (Iwao, 1968, 1972) [37,42]	β < 1, uniform distribution β → 1, random distribution β > 1, aggregated distribution
Taylor’s power method	$s^{2}=a{\bar{x}}^{b}$ → $\mathrm{lg}s^{2}=\mathrm{lg}a+b\mathrm{lg}\bar{x}$ (Taylor, 1961) [36]	b → 0, uniform distribution b → 1, random distribution b > 1, aggregated distribution

**Table 2:** Total sample density and aggregation index of *Spodoptera frugiperda* larvae in mountain maize fields in 2020.

Growth Stage	Mean Density per Plant	Variance (*S*^2^)	Mean Crowding Degree (m*)	Spread Index (*I*)	Patchiness Index (m*/m)	*C_a_* Index	Spread Coefficient (*C*)	*K* Value	Distribution Type
Seedling stage	0.27	1.12	3.41	3.14	12.62	11.62	4.14	0.09	Aggregation
Jointing stage	1.17	2.28	2.12	0.95	1.81	0.81	1.95	1.23	Aggregation
Trumpet stage	0.75	0.54	0.47	−0.28	0.63	−0.37	0.72	−2.68	Uniform distribution
Flare opening stage	0.35	0.49	0.74	0.39	2.12	1.12	1.39	0.89	Aggregation
Tasseling stage	0.08	0.07	0.00	−0.08	0.00	−1.00	0.92	−1.00	Uniform distribution
Powdering stage	0.12	0.13	0.17	0.05	1.39	0.39	1.05	2.57	Aggregation
Spinning stage	1.24	2.06	1.90	0.66	1.53	0.53	1.66	1.87	Aggregation
Pustulation stage	0.42	0.50	0.62	0.20	1.47	0.47	1.20	2.11	Aggregation
Milk ripening stage	0.05	0.05	0.00	−0.05	0.00	−1.00	0.95	−1.00	Uniform distribution

**Table 3:** Total sample density and aggregation index of *S. frugiperda* larvae in mountain maize fields in 2021.

Growth Stage	Mean Density per Plant	Variance (*S*^2^)	Mean Crowding Degree (m*)	Spread Index (*I*)	Patchiness Index (m*/m)	*C_a_* Index	Spread Coefficient (*C*)	*K* Value	Distribution Type
Seedling stage	1.39	4.91	3.91	2.52	2.81	1.81	3.52	0.55	Aggregation
Jointing stage	1.87	5.66	3.90	2.02	2.08	1.08	3.02	0.93	Aggregation
Trumpet stage	2.02	3.53	2.77	0.75	1.37	0.37	1.75	2.69	Aggregation
Flare opening stage	2.95	6.38	4.11	1.16	1.39	0.39	2.16	2.54	Aggregation
Tasseling stage	1.51	3.27	2.68	1.16	1.77	0.77	2.16	1.30	Aggregation
Powdering stage	0.58	0.52	0.48	−0.10	0.83	−0.17	0.90	−5.96	Uniform distribution
Spinning stage	2.76	7.34	4.42	1.66	1.60	0.60	2.66	1.67	Aggregation
Pustulation stage	2.50	3.21	2.78	0.29	1.12	0.12	1.29	8.66	Aggregation

**Table 4:** Theoretical sampling number of *S. frugiperda* under different densities of larvae in maize fields in 2020.

Larval Stage	Allowable Errors (*D*)	Larvae Density per Plant					
		0.5	1	1.5	2	2.5	3
Early-instar larvae	0.1	1632.0	910.1	669.4	549.1	476.9	428.8
	0.2	408.0	227.5	167.4	137.3	119.2	107.2
	0.3	181.3	101.1	74.4	61.0	53.0	47.6
Late-instar larvae	0.1	821.8	445.4	320.0	257.3	219.6	194.6
	0.2	205.4	111.4	80.0	64.3	54.9	48.6
	0.3	91.3	49.5	35.6	28.6	24.4	21.6
Total sample	0.1	1163.5	641.3	467.2	380.2	327.9	293.1
	0.2	290.9	160.3	116.8	95.0	82.0	73.3
	0.3	129.3	71.3	51.9	42.2	36.4	32.6

**Table 5:** The sequential sampling number of larvae of *S. frugiperda* in maize fields in 2020.

Larval Stage	Upper and Lower Limits	Number of Samples (*n*)														
		60	70	80	90	100	110	120	130	140	150	160	170	180	190	200
Early-instar larvae	upper limit *T*_1_(*n*)	32	37	41	45	49	53	57	61	65	69	73	77	81	85	89
	lower limit *T*_2_(*n*)	7	10	12	14	17	19	22	25	27	30	32	35	38	41	43
Late-instar larvae	upper limit *T*_1_(*n*)	28	32	36	40	44	48	51	55	59	63	66	70	74	78	81
	lower limit *T*_2_(*n*)	11	14	17	19	22	25	28	31	33	36	39	42	45	48	51
Total sample	upper limit *T*_1_(*n*)	30	34	39	43	47	51	55	58	62	66	70	74	78	81	85
	lower limit *T*_2_(*n*)	9	12	14	17	19	22	25	27	30	33	36	38	41	44	47

**Table 6:** Theoretical sampling numbers of *S. frugiperda* larvae in maize fields at different densities in 2021.

Larval Stage	Allowable Errors (*D*)	Larvae Density per Plant					
		0.5	1	1.5	2	2.5	3
Early-instar larvae	0.1	2612.1	1361.3	944.3	735.8	610.7	527.3
	0.2	653.0	340.3	236.1	184.0	152.7	131.8
	0.3	290.2	151.3	104.9	81.8	67.9	58.6
Late-instar larvae	0.1	743.9	359.6	231.5	167.4	129.0	103.4
	0.2	186.0	89.9	57.9	41.9	32.2	25.8
	0.3	82.7	40.0	25.7	18.6	14.3	11.5
Total sample	0.1	1259.8	694.6	506.2	412.0	355.5	317.8
	0.2	314.9	173.6	126.5	103.0	88.9	79.5
	0.3	140.0	77.2	56.2	45.8	39.5	35.3

**Table 7:** The sequential sampling number of *S. frugiperda* larvae in maize fields in 2021.

Larval Stage	Upper and Lower Limits	Number of Samples (*n*)														
		60	70	80	90	100	110	120	130	140	150	160	170	180	190	200
Early-instar larvae	upper limit *T*_1_(*n*)	36	40	45	49	54	58	62	66	71	75	79	83	87	91	95
	lower limit *T*_2_(*n*)	4	6	8	10	12	15	17	19	22	24	27	29	32	34	37
Late-instar larvae	upper limit *T*_1_(*n*)	28	32	36	40	44	48	52	56	59	63	67	71	74	78	82
	lower limit *T*_2_(*n*)	11	14	16	19	22	25	27	30	33	36	39	42	44	47	50
Total sample	upper limit *T*_1_(*n*)	31	35	39	43	47	51	55	59	63	67	71	75	78	82	86
	lower limit *T*_2_(*n*)	2	3	4	5	6	7	8	9	10	10	11	12	13	14	15

## Data Availability

The data presented in this study are available in article or supplementary file.

## Supplementary Materials

The following supporting information can be downloaded at: https://www.mdpi.com/article/10.3390/insects13100938/s1, Figure S1: Depth of pupation of *S. frugiperda* in mountain maize fields in 2020 and 2021; Table S1: The egg laying position and egg mass situation *S. frugiperda* on maize plants at jointing stage in 2021; Table S2: The investigation on damage part of the larvae of *S. frugiperda* on maize in mountain fields in 2020 and 2021; Table S3: Distribution of larvae *S. frugiperda* on maize plants in 2021; Table S4: Density and aggregation index of early-instar larvae of *S. frugiperda* in maize field in 2020; Table S5: Density and aggregation index of early-instar larvae of *S. frugiperda* in maize fields in 2021; Table S6: Density and aggregation index of late-instar larvae of *S. frugiperda* in maize field in 2020; Table S7: Density and aggregation index of late-instar larvae of *S. frugiperda* in maize fields in 2021.
