# Supplementary material for: Temporal and Spatial Distribution Patterns of Spodoptera frugiperda in Mountain Maize Fields in China
Source: Insects. 2022 Oct 16;13(10):938. doi: 10.3390/insects13100938 (PMC9604361; doi:10.3390/insects13100938)
Supplement: Supplementary file 1 [file insects-13-00938-s001.zip › insects-1898277-supplementary.pdf]

Supplementary file

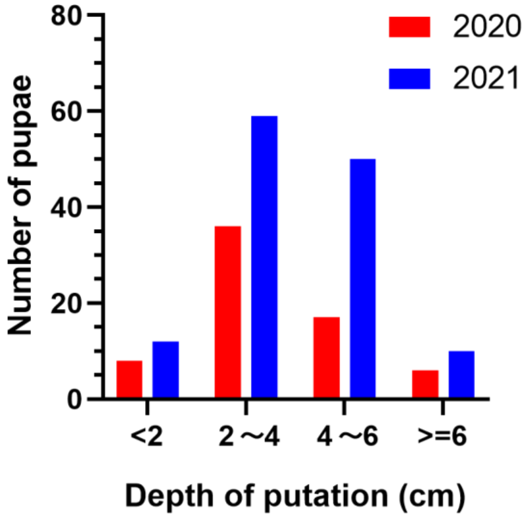

Figure S1. Depth of pupation of *S. frugiperda* in mountain maize fields in 2020 and 2021

Table S1. The egg laying position and egg mass situation *S. frugiperda* on maize plants at jointing stage in 2021

Egg mass location		Proportion (%)
Adaxial or abaxial leaves	Adaxial leaves	5.23
	Abaxial leaves	94.77
Distance from the midrib (cm)	0- 0.5	75.73
	0.5-1.0	18.83
	1.0-1.5	3.97
	≥1.5	1.46
Distance from main stem (cm)	0-10	46.96
	10-20	40.46
	20-30	8.18
	≥30	4.40
Egg grain number /mass	0-50	27.82
	50-100	42.68
	100-150	19.87
	≥150	9.62
Egg duration time（day）	1	25.94
	2	57.55
	3	16.51
With or without villus	Yes	48.95
	No	51.05
Parasitized or not	Yes	11.30
	No	88.70

Table S2. The investigation on damage part of the larvae of *S. frugiperda* on maize in mountain fields

Growth stage (Date)	Hazardous part
Seedling stage (6/15)	Heart and young leaves
Jointing stage (6/22)	Heart and young leaves
Trumpet stage (6/29～7/6)	Heart leaves
Flare opening stage (7/13～7/20)	Heart leaves
Tasseling stage (7/27)	Young leaves and tassel
Powdering stage (8/3)	Young leaves, tassel and ear
Spinning stage (8/10)	Ear
Pustulation stage (8/17～8/31)	Ear
Milk-ripe stage (9/7)	Ear
Wax-ripe stage (9/14)	Ear

Table S3. Distribution of larvae *S. frugiperda* on maize plants in 2021

Age	Relative distribution proportion (%)				Difference significance analysis
	Heart leaf	Stem (leaf)	Tassel	Ear	
1st instar	41.59±4.04	25.26±12.58	0	33.15±13.79	$F_{3,8}$=3.53, P=0.07
2nd instar	48.75±1.16a	21.85±1.30c	0.53±0.53d	28.87±0.30b	$F_{3,8}$=466.03, P<0.05
3rd instar	39.18±0.47a	20.53±2.64c	8.98±2.79c	31.32±0.77b	$F_{3,8}$=44.38, P<0.05
4th instar	34.31±3.07a	15.30±0.81b	12.35±0.90b	38.04±3.10a	$F_{3,8}$=33.17, P<0.05
5th instar	33.9±3.12a	15.84±0.30b	13.83±2.34b	36.44±2.03a	$F_{3,8}$=28.84, P<0.05
6th instar	24.51±2.74b	15.07±2.58b	18.62±4.17b	41.79±3.80a	$F_{3,8}$=12.25, P<0.05
1st-6th instar	37.73±1.37a	18.64±1.12b	9.13±1.08c	34.49±0.90a	$F_{3,8}$=169.39, P<0.05

Note: Values are mean ± SE. Data was analyzed by one way ANOVA with Tukey's HSD method at P<0.05. Different lowercase letters in the same line indicate significant differences.

Table S4. Density and aggregation index of early-instar larvae of *S. frugiperda* in maize field in 2020

Growth stage	Mean density plant^{-1}	Variance (S^2)	Mean crowding Degree (m^*)	Spread index (I)	Patchiness index （m^*/m）	*Ca* index	Spread coefficient (C)	*K* value	Distribution type
Seedling stage	0.20	1.06	4.50	4.30	22.50	21.50	5.30	0.05	Aggregation
Jointing stage	0.95	2.43	2.51	1.56	2.64	1.64	2.56	0.61	Aggregation
Trumpet stage	0.23	0.36	0.78	0.55	3.40	2.40	1.55	0.42	Aggregation
Flare opening stage	0.13	0.28	1.31	1.18	10.06	9.06	2.18	0.11	Aggregation
Tasseling stage	0.01	0.01	0.00	-0.01	0.00	-1.00	0.99	-1.00	Uniform distribution
Powdering stage	0.02	0.02	0.00	-0.02	0.00	-1.00	0.98	-1.00	Uniform distribution
Spinning stage	1.18	2.15	2.00	0.82	1.69	0.69	1.82	1.44	Aggregation
Pustulation stage	0.01	0.01	0.00	-0.01	0.00	-1.00	0.99	-1.00	Uniform distribution

$m^* = 0.88 + 1.49m$ (R = 0.44), $\alpha = 0.88 > 0$, $\beta = 1.49 > 1$;
$lgS^2 = 1.19lgm + 0.45$ (R = 0.98), $lga = 0.45 > 0$, $b = 1.19 > 1$

Table S5. Density and aggregation index of early-instar larvae of *S. frugiperda* in maize fields in 2021

Growth stage	Mean density plant^{-1}	Variance (S^2)	Mean crowding Degree (m^*)	Spread index (I)	Patchiness index （m^*/m）	*Ca* index	Spread coefficient (C)	*K* value	Distribution type
Seedling stage	1.39	4.91	3.91	2.52	2.81	1.81	3.52	0.55	Aggregation
Jointing stage	1.34	5.93	4.76	3.42	3.54	2.54	4.42	0.39	Aggregation
Trumpet stage	0.87	2.94	3.24	2.37	3.71	2.71	3.37	0.37	Aggregation
Flare opening stage	1.89	6.96	4.57	2.69	2.42	1.42	3.69	0.70	Aggregation
Tasseling stage	0.46	2.71	5.37	4.91	11.73	10.73	5.91	0.09	Aggregation
Powdering stage	0.09	0.18	1.00	0.91	10.71	9.71	1.91	0.10	Aggregation
Spinning stage	2.17	7.37	4.57	2.40	2.11	1.11	3.40	0.90	Aggregation
Pustulation stage	0.47	1.11	1.82	1.35	3.85	2.85	2.35	0.35	Aggregation

$m^* = 2.26 + 1.28744m$ (R = 0.62), $\alpha = 2.26 > 1$, $\beta = 1.29 > 1$;
$lgS^2 = 1.18lgm + 0.55$ (R = 0.9720), $a = 0.55 > 0$, $b = 1.18 > 1$

Table S6. Density and aggregation index of late-instar larvae of *S. frugiperda* in maize field in 2020

Growth stage	Mean density plant^{-1}	Variance (S^2)	Mean crowding Degree (m^*)	Spread index (I)	Patchiness index （m^*/m）	Ca index	Spread coefficient (C)	K value	Distribution type
Seedling stage	0.07	0.07	0.00	-0.07	0.00	-1.00	0.93	-1.00	Uniform distribution
Jointing stage	0.22	0.19	0.09	-0.13	0.41	-0.59	0.87	-1.70	Uniform distribution
Trumpet stage	0.52	0.36	0.21	-0.30	0.41	-0.59	0.70	-1.71	Uniform distribution
Flare opening stage	0.22	0.20	0.14	-0.08	0.62	-0.38	0.92	-2.63	Uniform distribution
Tasseling stage	0.07	0.07	0.00	-0.07	0.00	-1.00	0.93	-1.00	Uniform distribution
Powdering stage	0.10	0.11	0.20	0.10	2.00	1.00	1.10	1.00	Aggregation
Spinning stage	0.06	0.06	0.00	-0.06	0.00	-1.00	0.94	-1.00	Uniform distribution
Pustulation stage	0.41	0.49	0.60	0.18	1.44	0.44	1.18	2.25	Aggregation
Milk-ripe stage	0.05	0.05	0.00	-0.05	0.00	-1.00	0.95	-1.00	Uniform distribution

$m^* = -0.02 + 0.82m$ (R = 0.74), $\alpha = -0.02 < 0$, $\beta = 0.82 < 1$;
$lgS^2 = 0.97lgm - 0.55$ (R = 0.99), $lga = -0.55 < 0$, $b = 0.97 < 1$

Table S7. Density and aggregation index of late-instar larvae of *S. frugiperda* in maize fields in 2021

Growth stage	Mean density plant^{-1}	Variance (S^2)	Mean crowding Degree (m^*)	Spread index (I)	Patchiness index (m^*/m)	Ca index	Spread coefficient (C)	K value	Distribution type
Seedling stage	0.00	0.00	-	-	-	-	-	-	-
Jointing stage	0.53	0.56	0.58	0.05	1.09	0.09	1.05	10.90	Aggregation
Trumpet stage	1.14	0.96	0.99	-0.16	0.86	-0.14	0.84	-7.24	Uniform distribution
Flare opening stage	1.06	1.00	1.00	-0.06	0.95	-0.05	0.94	-18.30	Uniform distribution
Tasseling stage	1.06	0.94	0.94	-0.11	0.89	-0.11	0.89	-9.39	Uniform distribution
Powdering stage	0.49	0.42	0.36	-0.13	0.73	-0.27	0.87	-3.73	Uniform distribution
Spinning stage	0.60	0.65	0.69	0.10	1.17	0.17	1.10	6.05	Aggregation
Pustulation stage	2.02	1.87	1.95	-0.08	0.96	-0.04	0.93	-26.97	Uniform distribution

$m^* = 0.01 + 0.94m$ (R = 0.98), $\alpha = 0.01 < 1$, $\beta = 0.94 < 1$;
$lgS^2 = 0.93lgm - 0.03$ (R = 0.9825), $a = -0.03 < 0$, $b = 0.93 < 1$
